# Facile Synthesis of Gram-Scale Mesoporous Ag/TiO_2_ Photocatalysts for Pharmaceutical Water Pollutant Removal and Green Hydrogen Generation

**DOI:** 10.1021/acsomega.2c06657

**Published:** 2022-12-28

**Authors:** Yassine Cherif, Hajer Azzi, Kishore Sridharan, Seulgi Ji, Heechae Choi, Michael G. Allan, Sihem Benaissa, Karima Saidi-Bendahou, Lois Damptey, Camila Silva Ribeiro, Satheesh Krishnamurthy, Sanjay Nagarajan, M. Mercedes Maroto-Valer, Moritz F. Kuehnel, Sudhagar Pitchaimuthu

**Affiliations:** † Laboratoire de Catalyse et Synthèse en Chimie Organique, Université de Tlemcen, BP 119, Tlemcen13000, Algeria; ‡ Institut des Sciences et de la Technologie, Université d’Ain Témouchent, BP 284, 46000Ain Témouchent, Algeria; § Department of Nanoscience and Technology, School of Physical Sciences, University of Calicut, P. O. Thenhipalam673635, India; ∥ Theoretical Materials & Chemistry Group, Institute of Inorganic Chemistry, University of Cologne, Greinstr. 6, 50939Cologne, Germany; ⊥ Department of Chemistry, Swansea University, Singleton Park, SwanseaSA2 8PP, United Kingdom; # School of Engineering & Innovation, The Open University, Walton Hall, Milton KeynesMK7 6AA, United Kingdom; ∇ Department of Chemical Engineering, University of Bath, BathBA2 7AY, United Kingdom; ○ Research Centre for Carbon Solutions, Institute of Mechanical and Processing Engineering, School of Engineering & Physical Science, Heriot-Watt University, EdinburghEH14 4AS, United Kingdom; ◆ Fraunhofer Institute for Wind Energy Systems IWES, Am Haupttor 4310, 06237Leuna, Germany

## Abstract

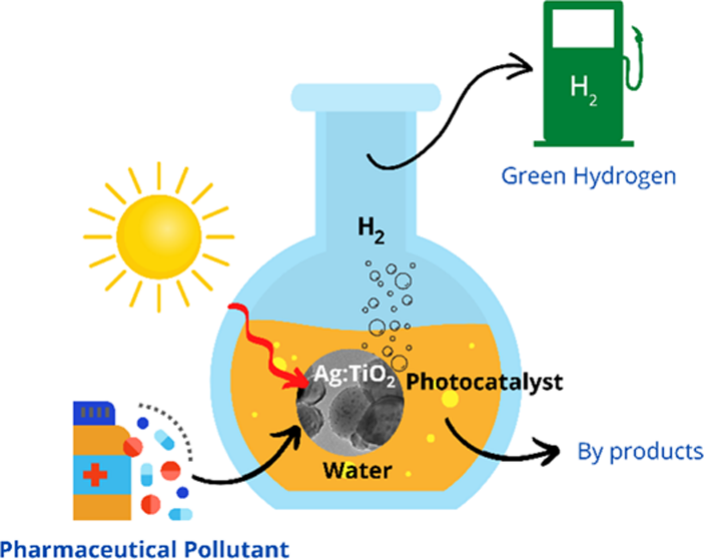

This work demonstrates a two-step gram-scale synthesis of presynthesized silver (Ag) nanoparticles impregnated with mesoporous TiO_2_ and evaluates their feasibility for wastewater treatment and hydrogen gas generation under natural sunlight. Paracetamol was chosen as the model pharmaceutical pollutant for evaluating photocatalytic performance. A systematic material analysis (morphology, chemical environment, optical bandgap energy) of the Ag/TiO_2_ photocatalyst powder was carried out, and the influence of material properties on the performance is discussed in detail. The experimental results showed that the decoration of anatase TiO_2_ nanoparticles (size between 80 and 100 nm) with 5 nm Ag nanoparticles (1 wt %) induced visible-light absorption and enhanced charge carrier separation. As a result, 0.01 g/L Ag/TiO_2_ effectively removed 99% of 0.01 g/L paracetamol in 120 min and exhibited 60% higher photocatalytic removal than pristine TiO_2_. Alongside paracetamol degradation, Ag/TiO_2_ led to the generation of 1729 μmol H_2_ g^–1^ h^–1^. This proof-of-concept approach for tandem pollutant degradation and hydrogen generation was further evaluated with rare earth metal (lanthanum)- and nonmetal (nitrogen)-doped TiO_2_, which also showed a positive response. Using a combination of *ab initio* calculations and our new theory model, we revealed that the enhanced photocatalytic performance of Ag/TiO_2_ was due to the surface Fermi-level change of TiO_2_ and lowered surface reaction energy barrier for water pollutant oxidation. This work opens new opportunities for exploiting tandem photocatalytic routes beyond water splitting and understanding the simultaneous reactions in metal-doped metal oxide photocatalyst systems under natural sunlight.

## Introduction

The increasing diversity of water pollutants has led to a growing global need to protect public health and the ecosystem.^1^ In particular, emerging pollutants such as pharmaceutical and personal care products (PPCPs) are a unique group of environmental contaminants due to their inherent ability to induce physiological effects in humans, even at low concentrations. These pollutants are becoming ubiquitous in the environment as they cannot be effectively removed by conventional wastewater treatment stages due to their toxicity and recalcitrance. Since these PPCPs may have adverse effects on humans and ecosystems, their eradication is of great interest in health and environmental risk management. To this end, advanced oxidation processes and especially semiconductor-based photocatalysis have recently gained increased attention to remove PPCPs due to their rapid degradation rates, cost-effectiveness, and mineralization capability.^2−4^

Under light irradiation, a valence band hole is created in a semiconductor photocatalyst alongside a photoexcited electron at the conduction band. These hole carriers lead to the formation of hydroxyl (^•^OH^–^) radicals; also, the photoelectrons at the conduction band generate superoxide (^•^O_2_^–^) and both are capable of degrading PPCP pollutants in water. Practically, TiO_2_ is the most active photocatalyst used in wastewater treatment owing to its high chemical stability, nontoxicity, high oxidation/reduction potential, and low cost.^5,6^ Recent reviews and research articles have emphasized the advantages of TiO_2_ photocatalyst-assisted pharmaceutical pollutant degradation.^7−9^ Mesoporous TiO_2_ has received much attention in photocatalytic wastewater treatment in recent times because it contains a highly interconnected pore network, which is favorable for the diffusion of reactants and products, and a large surface area, which offers more active sites.^10−12^ Owing to their excellent photocatalytic performance for pollutant oxidation^13−15^ and hydrogen generation,^16−18^ significant progress in synthesizing mesoporous TiO_2_ has been made in recent years. However, the quantum efficiency of mesoporous TiO_2_ is still not high enough (<1%) at present for practical applications. Many factors affect the photocatalytic activity of mesoporous TiO_2_, such as its specific surface area, crystallinity, etc. Various synthesis methods and modifications have been proposed to improve the photocatalytic performance of mesoporous TiO_2_, and their effects have been significant. Heteroatom doping effectively modifies TiO_2_ by introducing additional extrinsic electronic levels in the energy bandgap, thereby promoting visible-light absorption. For instance, metal doping into TiO_2_ creates intra-bandgap states due to the integration of metal dopants. Thus, the bandgap energy decreases mainly due to the lowering of the TiO_2_ conduction band. On the other hand, narrowing the bandgap at the desired level by incorporating nonmetal doping ions is even better. It creates oxygen vacancies (O_v_),^19^ which modify the electronic properties, surface chemistry, and coordination environment at TiO_2_, enhancing the visible-light activity and charge separation, thus promoting photocatalytic performance.^20,21^ Recently, a series of heteroatom dopants, including metal and nonmetal atoms (e.g., Cu, Ag, Au, La, N, C, etc.), have been reported to enhance the performance of mesoporous TiO_2_.^22−30^ In addition, reduced graphene oxide^31,32^ and graphene quantum dot (QD) composites^33,34^ also promoted charge transfer at TiO_2_ photocatalysts. The metal dopant dramatically increases the oxygen vacancy at the TiO_2_ lattice, modifies electron density, and reduces recombination between electron–hole pairs at the photocatalyst. Among different metals, silver (Ag) has a significant advantage of nontoxicity, lower cost, and higher antibacterial properties compared to its expensive counterparts (Au and Pt).^35−38^ Ag doping is commonly used to reduce the bandgap energy and minimize recombination by acting as an electron trap. Hence, Ag assists in charge separation by forming a Schottky barrier between TiO_2_ and metal. It also enhances the visible-light absorption of TiO_2_ due to the localized surface plasmon resonance (LSPR) originating from the oscillation of surface electrons.^39^

Recently, simultaneous pollutant degradation and hydrogen generation have received significant attention for addressing the energy demand and environmental clean-up concurrently.^40−43^ This dual process in one pot is expected to reduce the operating cost of both reactions carried out individually. Currently, there is little understanding of the influence of the photocatalyst surface properties on such tandem reactions, particularly regarding photocatalysts for concurrent pharmaceutical pollutant degradation and hydrogen generation. A major issue is high charge recombination rates at the photocatalyst surface severely affecting their performance. To address these challenges, this work explores the feasibility of material modification of the TiO_2_ photocatalyst in simultaneous water pollutant degradation and hydrogen generation.

The main objective of the work was to design high surface area mesoporous TiO_2_ coated with Ag nanoparticles by a two-step wet chemical synthesis process. The resultant Ag/TiO_2_ photocatalyst was tested for simultaneous paracetamol degradation and hydrogen gas recovery under natural sunlight. Furthermore, the tandem process was also evaluated with nonmetal (nitrogen)- and rare earth metal (Ln)-doped TiO_2_ photocatalysts. The origin of the photocatalytic performance of pristine TiO_2_ and Ag/TiO_2_ was examined with atomic-scale modeling using *ab initio* calculations and a Fermi-level-dependent adsorption energy theory model.

## Experimental Section

### Chemicals

Titanium butoxide (Sigma-Aldrich, 97%), PEG-PPG-PEG P123 (Sigma-Aldrich, 97%), acetic acid (Sigma-Aldrich, 97%), absolute ethanol (Honeywell Riedel-de Haën, 99.8%), silver nitrate (Sigma-Aldrich, 99%), sodium citrate tribasic dihydrate (Fluka Analytical, 99.0%), urea (Sigma-Aldrich, 99%), and lanthanum nitrate hexahydrate (Sigma-Aldrich, 99.999%) were used for the experiments. All of the chemicals were used as it is without filtering.

### Preparation of Bare and Ag-Doped Mesoporous TiO_2_

Mesoporous titania was prepared by the hydrothermal-assisted sol–gel method, as described by Liu et al.,^44^ with the following steps. First, *Solution A* was prepared by dropwise addition of 5 g of titanium butoxide into 30 mL of the 20% aqueous acetic acid solution under constant stirring for 4 h. Second, *Solution B* was obtained by dissolving 3 g of the block copolymer (Pluronic P123) in 20 mL of ethanol. In the third step, *Solution B* was added dropwise to *Solution A* and stirred at 150 RPM under room temperature for 24 h before crystallizing at 100 °C for 48 h in a Teflon-lined autoclave. After cooling the autoclave to room temperature, the solid product was collected by filtration, washed several times with distilled water, and dried in an oven at 80 °C overnight. Template removal was achieved by air calcination at 500 °C for 4 h with a ramping rate of 1 °C/min. The entire synthesis was carried out under standard atmospheric conditions.

Ag nanoparticle-coated mesoporous titania was synthesized by a simple chemical method developed by Naik et al.,^45^ in which AgNO_3_ is reduced by trisodium citrate. On previously prepared calcined mesoporous TiO_2_, different wt % (0.5 and 1) of Ag nanoparticles were introduced via the following procedure: an aqueous solution containing an appropriate amount of AgNO_3_ was heated at 80 °C for 20 min in a round-bottomed flask. Then, 0.5 g of mesoporous titania was added to this solution and kept under stirring (150 RPM) for 30 min. The 0.002 M trisodium citrate aqueous solution was then added dropwise to the mixture. After 1 h of stirring (150 RPM), the solution was cooled to room temperature, filtered and washed five times with 60 °C deionized water, and then dried in a vacuum oven at 60 °C for 60 h. The photographs of pure and Ag nanoparticle-coated TiO_2_ powders at the gram scale are presented in Figure S1.

### Preparation of N- and La-Doped Mesoporous TiO_2_

The photocatalytic performance of Ag/TiO_2_ was compared against other doped titania. First, nitrogen-doped mesoporous titania with N/TiO_2_ molar ratio equal to 3 was prepared as reported by Jian et al.,^46^ in which urea was used as the nitrogen source and mixed with TiO_2_ in an agate mortar. The resulting mixture was calcined at 500 °C (10 °C/min) for 1 h. The photocatalyst is denoted as N/TiO_2_ from hereon. In the case of lanthanum (La)-doped TiO_2_, 2 wt % of lanthanum was impregnated on calcined mesoporous TiO_2_ by the incipient wet impregnation technique using lanthanum nitrate as the La source. TiO_2_ was mixed with the 0.002 M aqueous solution of lanthanum nitrate at 60 °C in a rotary evaporator. Then, the impregnated powder was dried at 100 °C overnight and calcined in air at 500 °C for 4 h (heating rate: 1 °C/min).

### Catalyst Characterization

The X-ray diffraction (XRD) patterns were recorded using a Rigaku MiniFlex 600 diffractometer equipped with monochromatized Cu Kα radiation (λ = 15,418 Å). The morphology of the powder samples was characterized by high-resolution transmission electron microscopy (HRTEM, JEM2000EX). X-ray photoelectron spectroscopy (XPS) spectra were performed with a Kratos XSAM 800 kit having a dual anode X-ray source. The N_2_ adsorption/desorption isotherms were carried out on a 3Flex instrument (Micromeritics). The specific surface areas of the samples were calculated by the BET method. The UV–vis diffuse reflectance spectra of the photocatalysts were recorded on a SPECORD 200 Plus UV–vis spectrophotometer. Raman spectra were recorded on a UV resonance Raman spectrometer (Horiba LabRAM HR Evolution Raman spectrometer). The laser excitation was at 633 nm.

### Photocatalytic Paracetamol Degradation

The photocatalytic activity of pristine TiO_2_ and Ag/TiO_2_ was assessed by observing the degradation of paracetamol under natural solar light irradiation (latitude: 34°52′41.99″N; longitude: −1°18′54.00″W) on June 2021 from 12 to 2 pm as shown in Figure S2. The outside temperature was 28 °C, whereas the experimental solutions reached 36 °C. The reaction slurry was prepared by suspending 100 mg of the photocatalyst in 100 mL of an aqueous paracetamol solution (10 mg/L). The slurry was stirred in the dark for 30 min to ensure the adsorption of paracetamol molecules on the surface of the photocatalysts. During the photocatalytic reaction, aliquots (5 mL) of the reaction slurry were withdrawn at regular intervals of time, centrifuged (4500 RPM for 10 min) to separate the photocatalyst, and the supernatant was stored in amber glass vials. To determine the performance variability, all experiments were conducted in triplicates. The rate of the photocatalytic degradation of paracetamol was determined from the absorption spectra of the centrifuged aliquots that were measured using a UV–vis spectrophotometer (SPECORD 200 PLUS) and were compared with those of the original solution. A decrease in the absorbance of paracetamol with respect to the irradiation time was used to determine the efficiency of the photocatalysts. Total organic carbon (TOC) analysis was carried out for the most active catalyst with an Analytik Jena multi N/C 3100 analyzer.

#### Photocatalytic H_2_ Evolution: Sample Preparation

TiO_2_ powder (5.0 mg, unless otherwise stated) was transferred into a glass sample vial (Chromacol 10-SV, Fisher) along with the reagent solution containing 0.1 M triethanolamine (TEOA) with pH 7.0 (3.0 mL, unless otherwise stated). Samples were capped with rubber septa, briefly vortexed, and agitated in a sonic bath for 20 min. Samples were purged with N_2_ for 10 min prior to irradiation to deaerate the solution. Samples were irradiated using a solar light simulator (Thermo Oriel 92194-1000) equipped with an AM 1.5G filter (Newport) with an intensity of 1 sun. Samples were mounted in a quartz water bath maintained at 25 °C and stirred at 800 RPM. The sample headspace was subject to a constant purge of N_2_ at a rate of 4 mL min^–1^ controlled by a mass flow controller (Bronkhorst). H_2_ evolution was monitored by the online gas chromatographic analysis of the headspace stream.

#### Sample Analysis by Gas Chromatography

A Shimadzu Nexis GC-2030 gas chromatograph equipped with a barrier-discharge ionization detector (BID) and a molecular sieve column (5A PLOT capillary column, 30 m × 0.53 mm, 50 μm, kept at 140 °C) was used to quantify hydrogen produced in the process. The total run time of the method was 5 min. The GC was calibrated using calibration gas (2000 ppm H_2_, BOC), diluted with N_2_ at different ratios using a set of mass flow controllers (Bronkhorst) to provide known concentrations of H_2_. Gas samples were programmed to auto-inject into the GC via a multiport stream selector valve directing the selected sample purge gas stream through a 2 mL sample loop before injection. H_2_ evolution rates were calculated from the measured H_2_ concentration in the purge gas and the purge gas flow rate. Cumulative H_2_ production was calculated from the H_2_ evolution rate and time elapsed since the previous measurement, assuming a constant H_2_ evolution rate between time points. All analyses were performed in triplicates unless otherwise stated.

### Computational Modeling

All density functional theory (DFT) calculations were performed by the projector-augmented wave method (PAW) implemented in the Vienna ab initio Simulation Package (VASP).^47−49^ The Perdew–Burke–Ernzerhof (PBE) function within the generalized gradient approximation (GGA) was used to describe the exchange–correlation function.^50,51^ A cut-off energy of 400 eV was used for the plane wave basis set. The energy and force convergence criteria for geometry optimization were set as 10^–6^ eV and 0.02 eV/Å, respectively. The Brillouin zone was sampled with a 2 × 2 × 1 Monkhorst–Pack *k*-point mesh.^52^ The optimized lattice parameters of TiO_2_ (001) and TiO_2_ (101) are 11.36 Å × 11.36 Å × 13.17 Å and 10.92 Å × 15.14 Å × 9.81 Å, respectively. The vacuum space is larger than 10 Å to avoid an interlayer interaction. The onsite Coulombic interaction corrections approach (DFT + *U*) with *U* = 5 eV was employed to treat the 3d orbital electrons of Ti atoms.^53^

Recently, Kim et al.^54^ experimentally and theoretically demonstrated that the adsorption energy of rutile TiO_2_ can be tuned by a changeable Fermi level resulting in charged intermediates during chemisorption. In this regard, the Gibbs free energies of OER intermediates on an anatase TiO_2_ (101) surface in this work were calculated using the following equation
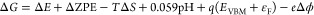
1where Δ*E*, ΔZPE, and Δ*S* are the adsorption energies, zero-point energies, and entropy difference, respectively, ε_F_ and *E*_VBM_ are the Fermi level and DFT-computed eigenvalue of the VBM energy level of anatase TiO_2_ (101), respectively, and Δ*ϕ* in the last term of eq 1 is the potential difference between the valence band maximum (VBM) and the water oxidation potential, which is added to take the potential energy change of photogenerated holes during oxidation.

## Results and Discussion

**Figure 1:**
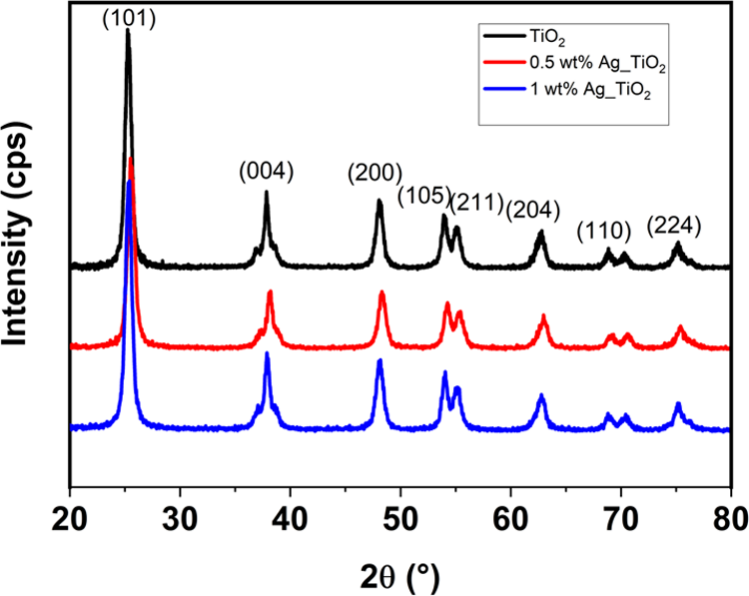
X-ray diffraction analysis of pristine and Ag nanoparticle-coated TiO_2_ powders.

XRD analysis was performed to investigate the structural properties of the pristine TiO_2_ and Ag/TiO_2_ samples. As seen in Figure 1, the peaks of all of the samples at 2θ values of 25.5, 37.8, 48.0, 53.9, 55.1, 62.7, 68.8, 70.3, and 75.0° match the reflections from (101), (004), (200), (105), (211), (204), (116), (220), and (215) crystal planes of the tetragonal anatase phase of TiO_2_ (JCPDS card no. 21-1217) with lattice constants *a* = 3.785 Å and *c* = 9.513 Å.^4^ Despite the addition of Ag metal (0.5 and 1 wt %) as implants/dopants, the XRD patterns of the modified TiO_2_ samples appeared similar to that of pristine TiO_2_. This can be attributed to the small weight percentage of externally added atoms leading to a well-dispersed mix in the TiO_2_ matrix, thereby unaffecting the crystalline structure.

**Figure 2:**
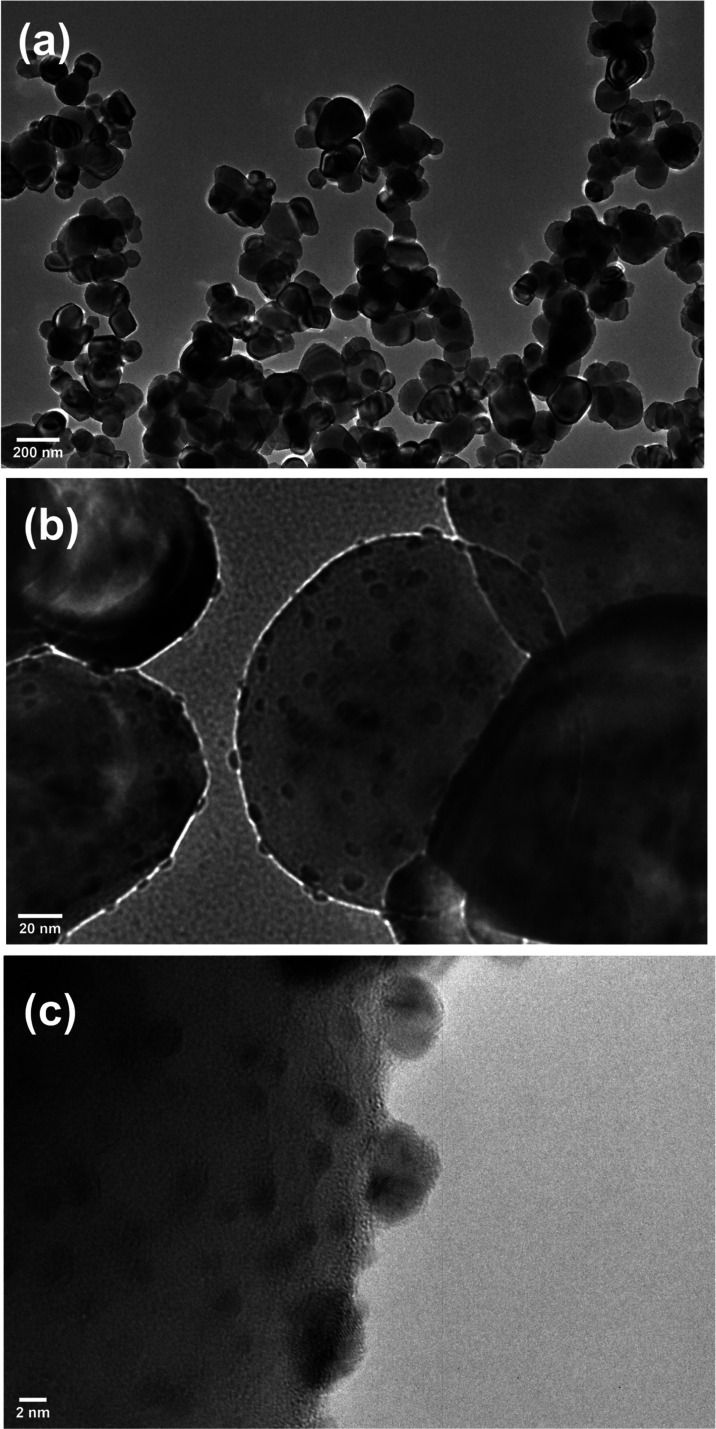
HRTEM images of the Ag (1 wt %) nanoparticle-coated TiO_2_ powder at different magnification scales (a) 200 nm, (b) 20 nm, and (c) 2 nm.

The morphology and microstructure of the as-synthesized mesoporous pristine and doped TiO_2_ were analyzed through TEM images. The TEM images of pure TiO_2_ in Figure S3a showed that the TiO_2_ particles were in the 10–15 nm size range, and these particles appear to be clustered together, which is confirmed by the high-resolution TEM micrograph shown in Figure S3a. Further, the clearly appearing lattice indicates the crystalline nature of the particles with a spacing of 3.5 Å, which corresponds to the (101) plane of tetragonal anatase TiO_2_Figure S3b. Upon Ag nanoparticle deposition on TiO_2_, the particle size and shape of the TiO_2_ host significantly changed (Figure 2a). The particle size enhancement of TiO_2_ from 10–15 to 80–100 nm might be due to the presence of inorganic moieties in the Ag nanoparticle solution. Mainly the traces of trisodium citrate may influence the TiO_2_ particle growth. However, the origin of TiO_2_ particle size enhancement is not clear. The Ag nanoparticles are randomly distributed on the TiO_2_ surface, which leaves naked sites at TiO_2_. These uncoated TiO_2_ sites can allow access to both Ag and TiO_2_ surface sites for photocatalytic reactions. Figure S4 shows that the (111) plane of face cubic center (FCC) Ag is clearly observed on the TiO_2_ surface.

**Figure 3:**
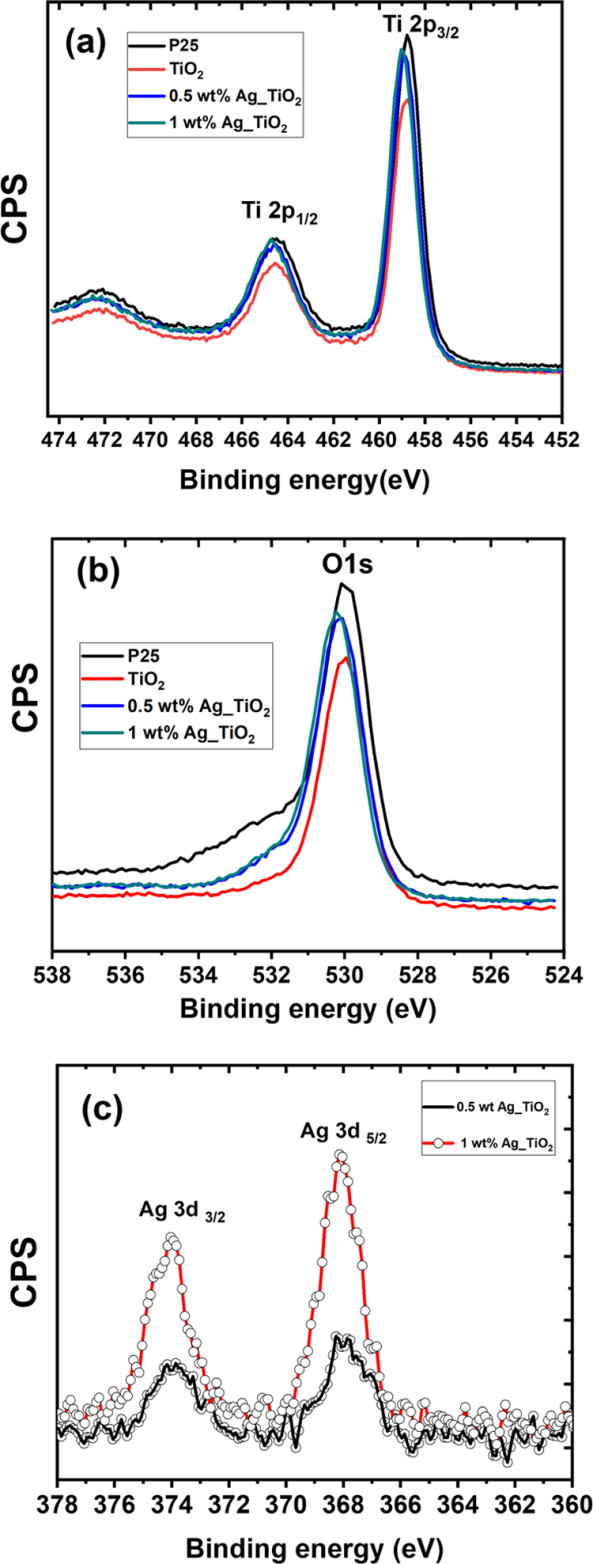
XPS core spectra of (a) Ti 2p, (b) O 1s, and (c) Ag 3d. The results of panels (a and b) are compared with those of the commercial P25 powder.

The chemical environment of pristine TiO_2_ and Ag nanoparticle-coated TiO_2_ was studied with XPS spectra (Figure 3a,b). Figure 3a,b shows the high-resolution XPS core spectra of Ti 2P and O 1S of pristine and nanoparticle-coated mesoporous TiO_2_. In Figure 3a, Ti 2p_3/2_ and Ti 2p_1/2_ peaks are observed at binding energies of 458.7 and 464.5 eV, respectively. The difference in these binding energies is 5.8 eV which corresponds to the +4 oxidation state in mesoporous TiO_2_.^55−57^ The O 1s peak at 530 eV is perfectly symmetric without any shoulder at higher binding energies, suggesting the absence of different oxygen species in the mesoporous TiO_2_. The binding energies of Ti 2p_3/2_, Ti 2p_1/2_, and O 1s peaks are in accordance with those reported for anatase TiO_2_,^58^ which is in good agreement with the XRD results. The XPS results of mesoporous TiO_2_ were also compared with commercial P25 TiO_2_ (Figure 3a,b). Figure 3b indicates that commercial P25 TiO_2_ has a broader peak at 532.2 eV attributed to hydroxyl groups on the surface, which help to adsorb the water pollutants on the TiO_2_ surface during the photocatalysis reactions. Interestingly, the peak shoulder broadening at 532.2 eV is missing in mesoporous TiO_2_.

The Ag 3d high-resolution XPS spectra of Ag nanoparticle-coated mesoporous titania are depicted in Figure 3c. The Ag 3d_5/2_ and Ag 3d_3/2_ peaks are observed at binding energies of 368.1 and 374 eV, which we attribute to Ag^0^.^59,60^ Note that these two peaks are broad and could overlap with peaks attributed to Ag_2_O and the electronic interaction between the metal and support. For Ag_2_O, the binding energies of the Ag 3d_5/2_ and Ag 3d_3/2_ peaks are observed at 367.73 and 373.71 eV.^61^ Gogoi et al.^37^ attributed the lower binding energies of Ag 3d_5/2_ (366.45 eV) and Ag 3d_3/2_ (372.5 eV) to the electronic interaction between the metal and support by charge transfer at the metal–support interface. The Ag 3d peak intensities understandably increased with increasing Ag loading. It can be seen from Figure 3a that Ti 2p spectra shift to higher binding energies upon silver doping. We ascribe this shift to an increase in the effective positive charge of Ti owing to the electronic redistribution caused by the dopant, leading to a decrease in the Ti outer electron density, a reduction in the shielding effect, and an increase in the electron binding energy.^62,63^ These effects are likely beneficial for enhancing photocatalytic activity. The O 1s spectra of Figure 3b show the appearance of the shoulder located at a binding energy of 532.2 eV after silver doping, which is attributed to OH groups on the surface.^64^ Hydroxyl groups (OH^–^) on the surface of the catalyst positively affect the photocatalytic activity.

With the increasing hydroxyl content on the surface of TiO_2_, the surface becomes more likely to enhance the photocatalytic activity of TiO_2_.^62^ On the other hand, the increase in surface OH^–^ content could promote electron–hole separation, increasing the photocatalytic activity.^65,66^ Further analyzing the C 1s spectra of Ag-coated TiO_2_ (Figure S5) shows an increased intensity of the peak at a binding energy of 289.7 eV compared with pure TiO_2_, which could be ascribed to the presence of citrate (reducing agent) adsorbed on silver nanoparticles, which is in good agreement with Raman characterization results (Figure S6).

**Figure 4:**
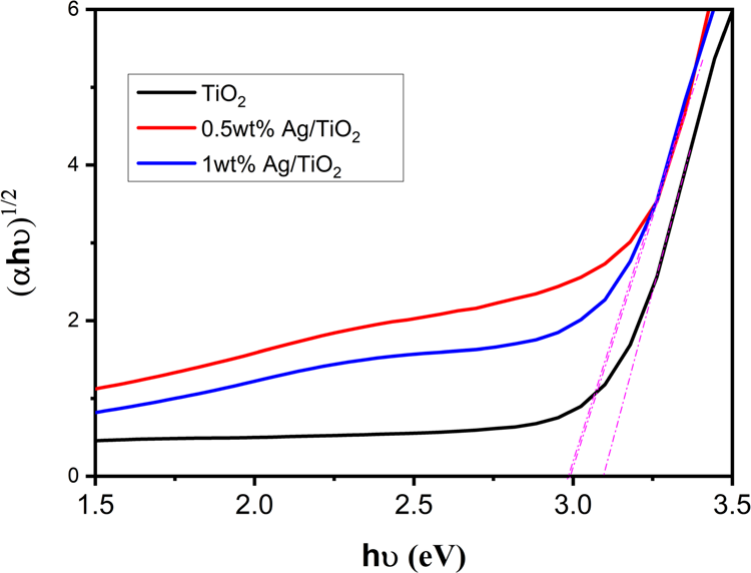
Plots of the Kubelka–Munk function versus the energy of the absorbed light for pure and Ag nanoparticle-coated mesoporous TiO_2_.

The optical bandgap of pristine and Ag/TiO_2_ was estimated from UV–vis diffuse reflectance spectra given by (α*h*υ)^1/*n*^ = *A*(*h*υ – *E*_g_), where *E*_g_ is the optical bandgap energy, α is the absorption coefficient, *h* is the Planck′s constant, υ is the frequency of light, *A* is the proportionality constant and *n* are 1/2 and 2, respectively for direct and indirect bandgap semiconductors. Since TiO_2_ is an indirect bandgap semiconductor, a plot between photon energy *h*υ and (α*h*υ)^1/2^ was constructed, and *E*_g_ was estimated by extrapolating the linear portion of the y-axis onto the *x*-axis, as shown in Figure 4. The bandgap energy of pure TiO_2_ is reduced from 3.1 to 2.91 eV by Ag nanoparticle coating.

**Figure 5:**
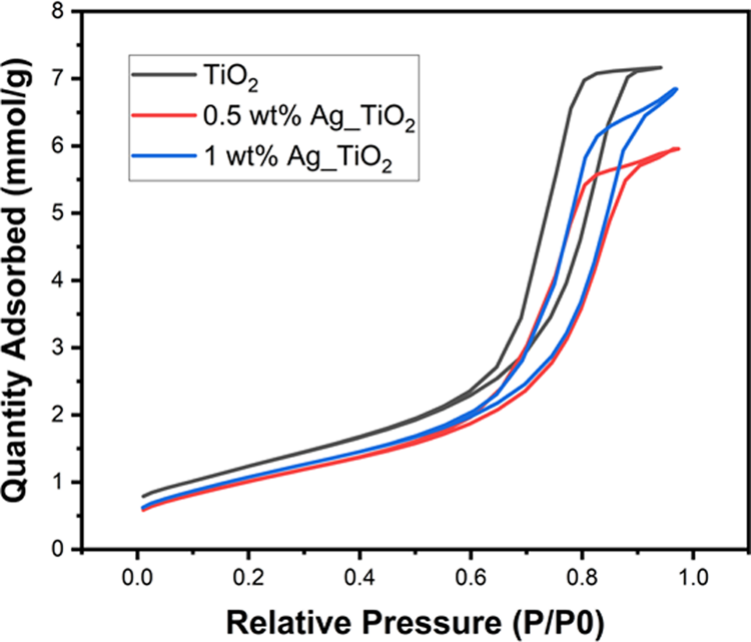
Adsorption–desorption isotherms of nitrogen on pristine and Ag nanoparticle-coated mesoporous TiO_2_ at 77 K.

The porosity of different photocatalysts was studied by BET analysis. Figure 5 shows typical irreversible type IV N_2_ adsorption isotherms with an H1 hysteresis loop^67^ for pristine and Ag/TiO_2_. The surface area and pore volume of the pure and Ag/TiO_2_ are presented in Table S1. It is worth noting that the mesoporous TiO_2_ synthesized in this work exhibited a high specific surface area of 102 m^2^/g, which is 2.5 times higher than that of commercial P25 TiO_2_ (56 m^2^/g).^68,69^ Also, it resulted in a significantly higher pore volume of 0.325 cm^3^/g compared with commercial P25 TiO_2_ (0.02 cm^3^/g).^68^ It is inferred that the P123 surfactant templates effectively induced the mesoporous network at TiO_2_, which is the reason for the increased surface area compared with the commercial P25 TiO_2_ powder. As can be seen from Table S1, the textural properties of pure mesoporous TiO_2_ are maintained, whatever the mass percentage of Ag nanoparticle coating. The slight decrease in the specific surface area after doping with Ag is ascribed to the clogging of support pores by silver that makes them inaccessible for nitrogen adsorption.^70^

**Figure 6:**
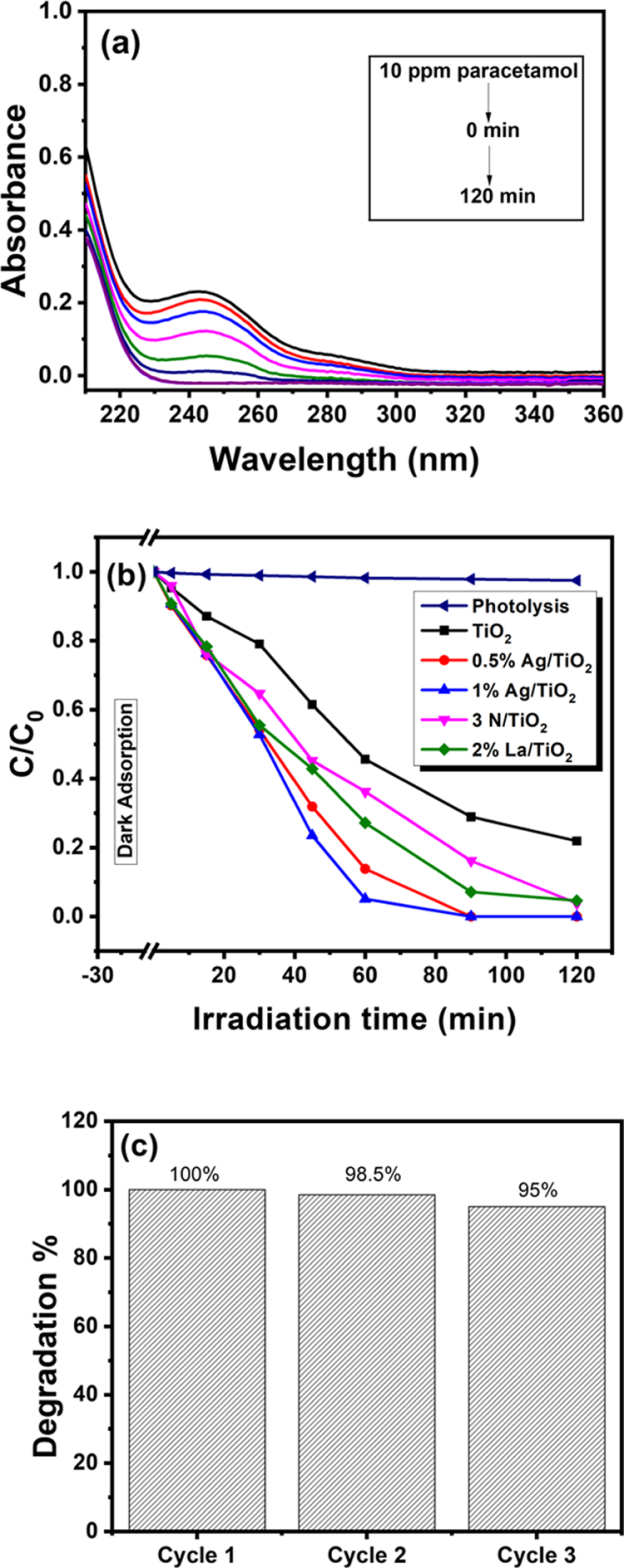
(a) UV–Visible absorption spectra of paracetamol photodegradation using 1 wt %Ag/TiO_2_ and (b) paracetamol photocatalytic degradation over pristine and Ag nanoparticles-coated mesoporous TiO_2_ under natural solar light irradiation. The results were compared with N- and La-doped TiO_2_ powder samples. (c) Recycling photocatalytic degradation of paracetamol over 1 wt % Ag/TiO_2_ under natural solar light irradiation.

The photocatalytic activity of pristine TiO_2_ and Ag/TiO_2_ photocatalysts was evaluated through the photocatalytic degradation of paracetamol in water under natural sunlight. The experimental setup is shown in Figure S2. UV–vis absorption spectra corresponding to the visible-light-driven photocatalytic degradation of paracetamol in the presence of 1 wt % Ag/TiO_2_ are presented in Figure 6a, wherein the consistent reduction in the intensity of the characteristic absorption peak (244 nm) of paracetamol is indicative of the decrease in its concentration. The *C*/*C*_0_ values for all of the photocatalyst samples are estimated and presented in Figure 6b as a function of irradiation time. As seen in Figure 6b, the photodegradation of paracetamol was negligible under visible-light irradiation only in the absence of any photocatalyst. Figure 6b shows that the absorbance of the paracetamol solution (10 mg/L) after stirring with TiO_2_-based photocatalysts in the dark for 30 min is nearly constant. This indicates that the adsorption of paracetamol on the catalyst surface is negligible due to the neutrality of the paracetamol molecule. The degradation performance of Ag/TiO_2_ was compared against N- and La-doped TiO_2_, as shown in Figure 6b, to investigate the effect of other dopants (nonmetal and rare earth elements) on the photocatalytic performance of mesoporous TiO_2_. Note that the N- and La-doping at TiO_2_ are not identical in quantity to Ag nanoparticles. The 2 wt % La-doped mesoporous TiO_2_ resulted in higher degradation of paracetamol compared to pristine TiO_2_. Earlier studies have reported that La-doping increased the adsorption capacity of organic compounds and inhibited the e^–^–h^+^ recombination during the photocatalytic reaction.^71,72^

In the case of N-doped TiO_2_, the improved photocatalytic activity compared to pristine TiO_2_ resulted from the slightly extended absorption in the visible-light range, indicating that more photogenerated electrons and holes can participate in the photocatalytic reactions under visible light^73−75^ (Figure S6). The improvement of visible-light absorption after nitrogen doping can be attributed to bandgap narrowing, the creation of an impurity energy level, or even oxygen vacancies.^76,77^

In general, the toxic organic molecules can be degraded via photocatalysis, but their byproducts may require additional time to completely degrade into nontoxic minerals, which is safer for discharge into water bodies.^78^ Analyzing changes to the total organic carbon (TOC) in the reaction system ensured the complete removal of toxic organic molecules.^78^ Therefore, the photocatalytic mineralization of paracetamol using 1 wt %Ag/TiO_2_ was evaluated. It implied that, although 10 ppm paracetamol was completely degraded (∼100%) within 120 min, the intermediate organic byproducts took up to 300 min to mineralize (98%, as measured using a TOC analyzer). In addition to higher photocatalytic efficiency, the stability of the photocatalyst against photocorrosion is a crucial factor that is usually considered for deciding its employability for industrial applications. Therefore, for studying the effect of photocorrosion on 1 wt % Ag/TiO_2_, the photocatalytic degradation of paracetamol was performed repeatedly for three continuous cycles by reusing the catalyst after its separation from the residual slurry through centrifugation. As evident from Figure 6c, there is an insignificant decline in the photodegradation efficiency, which could be attributed to the loss of the photocatalyst during each round of centrifugation and rinsing.

**Figure 7:**
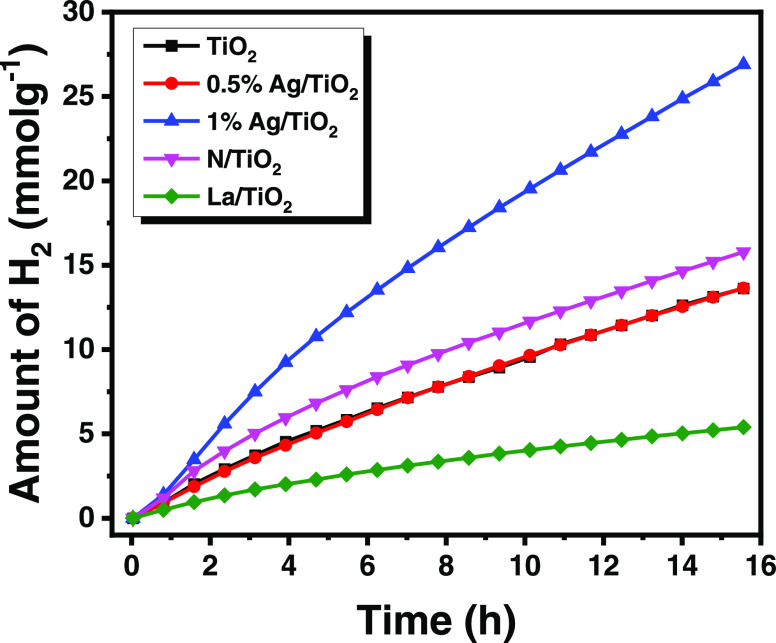
Photocatalytic H_2_ production using different photocatalysts measured at 1 sun irradiation. Note that the electrolyte contains paracetamol (10 ppm) and TEOA, a sacrificial agent.

Due to technical difficulties in outdoor sunlight irradiation experiments for measuring the hydrogen gas generation via gas chromatography, we demonstrated H_2_ gas generation indoors using simulated solar irradiation (AM 1.5 G). The aqueous paracetamol (10 ppm)-based electrolyte similar to the above experiment was tested in photocatalytic hydrogen generation reactions, but there is no hydrogen generation observed, which may be due to the inadequate concentration of paracetamol needed to produce donors or the slowest oxidation rate of paracetamol is not enough to produce H^+^. Therefore, we added an organic sacrificial agent (TEOA) along with paracetamol, experiments were repeated with different photocatalysts, and the corresponding hydrogen gas generation was measured for 14 h. The results are presented in Figure 7. It can be seen from this figure that the H_2_ production is almost linear with the reaction time. We expected paracetamol degradation alongside hydrogen gas evolution to be possible, but we did not verify it.

For the 1 wt % Ag/TiO_2_ catalyst, the H_2_ production rate was exalted after 3 h of reaction. The photocatalytic activity of the samples varies in the following decreasing order: 1 wt %Ag/TiO_2_ > 0.5 wt %Ag/TiO_2_ > TiO_2_. It is interesting to note that 1 wt % Ag/mesoporous TiO_2_ is the most active catalyst for both the photodegradation of paracetamol and H_2_ production. The highest photocatalytic hydrogen evolution activity achieved was 1729 μmol H_2_ g^–1^ h^–1^, which largely exceeded that obtained over bare mesoporous TiO_2_ (875 μmol H_2_ g^–1^ h^–1^). On the other hand, for the other catalysts, the ranking of activities is different from that obtained for the photodegradation of paracetamol.

Note that 2 wt % La/TiO_2_ was less active than TiO_2_. Liu et al.^79^ reported that the proper amount of lanthanum-doped TiO_2_ enhanced the photocatalytic hydrogen production, but excessive lanthanum ions inhibited the activity by blocking active sites on TiO_2_. In our case, we cannot advance this explanation since 2 wt % La/TiO_2_ was more active than TiO_2_ for paracetamol degradation. The negative effect of lanthanum could be explained by the fact that lanthanum shifts the conduction band maximum (CBM) below the H^+^/H_2_ reduction potential, which means that it does not meet the requirement for H_2_ evolution.^80,81^ This result is in contradiction with those of Shwetharan et al., who used La-TiO_2_ prepared by direct synthesis using La_2_O_3_ as the lanthanum source.^82^ In our study, lanthanum is not incorporated into the lattice of TiO_2_, unlike what was reported by Shwetharan et al., which could explain this contradictory result. In the case of N doping, TiO_2_ performed better for hydrogen evolution due to the enhancement of visible-light activity at TiO_2_. But its paracetamol degradation performance was inferior to La-doped TiO_2_, suggesting that its valence band position may be less positive than the pollutant oxidation potential.

**Figure 8:**
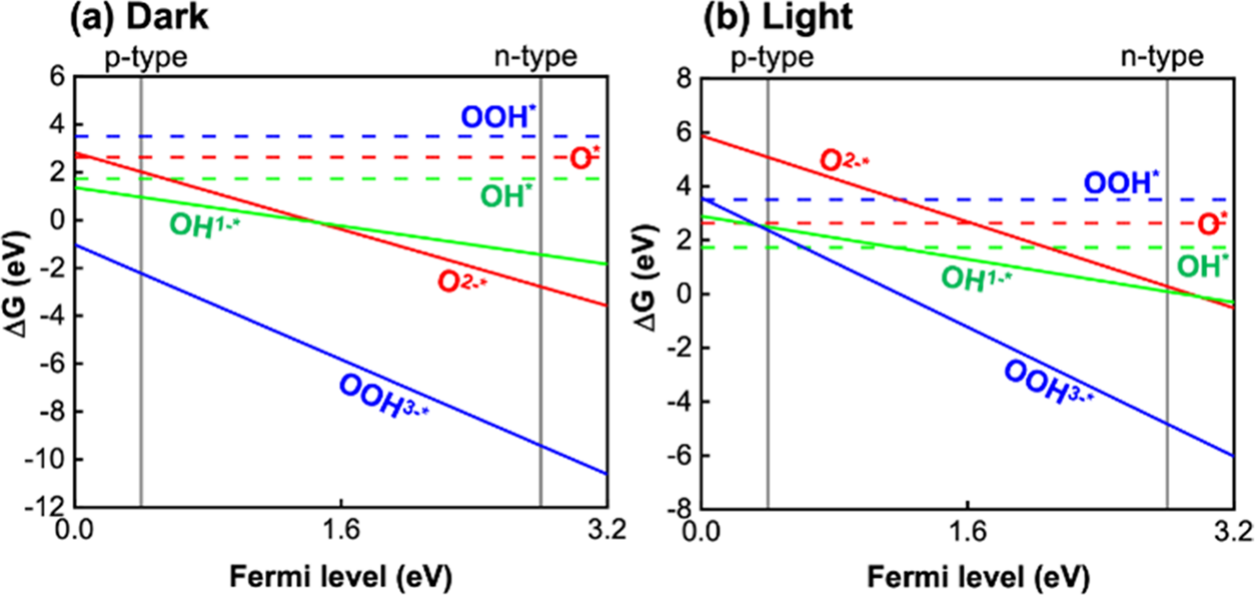
Gibbs free energy graph as a function of the Fermi level of anatase TiO_2_ (101) for OER at pH 7 under (a) dark and (b) light irradiation conditions.

Overall, we proved the suitability of Ag nanoparticle-coated mesoporous TiO_2_ photocatalysts for simultaneous pharmaceutical water pollutant degradation and hydrogen generation. The results presented in Figures 6b and 7 show paracetamol degradation and hydrogen gas evolution on the same Ag/TiO_2_ photocatalyst. To understand the effect of Ag metal loading on the anatase TiO_2_ (101) surface on the catalytic reactivity of the oxygen evolution reaction (OER) process at pH 7 under UV light irradiation, we calculated Gibbs free energies of OER intermediates using eq 1 (Figure 8). We considered that the adsorbate intermediates of OER (OH*, O*, and OOH*) can be charged due to the charge transfer between the anatase TiO_2_ (101) surface and the adsorbates. All possible charge states of adsorbates were considered for modeling and the Gibbs free energy calculations.

**Figure 9:**
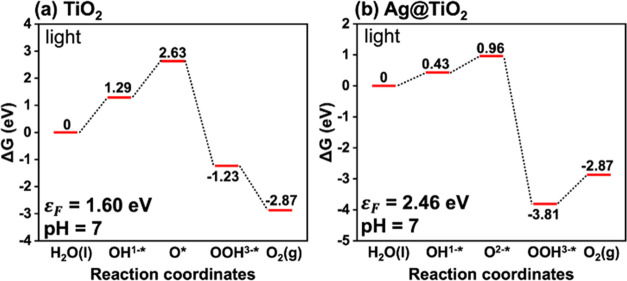
Gibbs free energy diagrams of (a) TiO_2_ and (b) Ag@TiO_2_ under UV light irradiation.

Then, we compared two cases of surface kinetic energies (Gibbs free energy diagram): anatase TiO_2_ (i) without Ag metal loading (ii) with Ag metal loaded on the surface (Ag@TiO_2_) (Figure 9). Considering that the typical upward band bending of an n-type semiconductor is nearly 1 eV and the average Fermi level of anatase TiO_2_ lies at 2.6 eV,^83^ the surface Fermi level of bare TiO_2_ was assumed to be 1.6 eV (Figure 9a). In general, the work function of a metal is a decisive factor for the band bending at the interface of a metal–semiconductor heterojunction. The difference in the work function between pristine n-type anatase TiO_2_ (∼4.7 eV) and Ag (4.74 eV) is tiny (<0.1 eV) compared to the band bending energy of the bare anatase TiO_2_(101) surface (∼1 eV) induced by the space charge of TiO_2_.^83−85^

Therefore, the surface Fermi levels of the two materials are aligned at the equilibrium state, resulting in band bending at the interface. According to the Gibbs free energy graph, relevant reactive intermediates in the water oxidation mechanisms are OH*, O*, and OOH* intermediates, where * indicates the surface-adsorbed states of OH, O, and OOH. The reaction steps can be explained as^86,87^
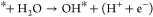
2
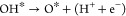
3
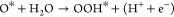
4
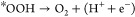
5

The reactive intermediate O* can be classified as dangling O* (O1*) and surface-bound peroxo species (O2*). The stability of O* depends on the semiconductor catalyst.^87^ In accordance with Malik et al.,^86^ when Δ*G*_OH*_ > 2.73 eV, the photocatalyst will produce ^•^OH in the electrolyte via the one-electron transfer process. Also, they suggested that peroxo O2* intermediate species are more stable than dangling O1* species, which is in favor of the two-electron process to form H_2_O_2_. Furthermore, the product ratio and selectivity (H_2_O_2_ vs. O_2_) will be dictated by the kinetic barriers rather than thermodynamic applied potentials.^86^ From Figure 9(b), the energy barrier in the rate-determining steps of the OER process was lowered at Ag/TiO_2_ (ε_F_ = 2.46 eV) by 0.86 eV in comparison to that of bare anatase TiO_2_ (ε_F_ = 1.60 eV). Ultraviolet photoelectron spectra (Figure S7) show that the CB maximum position changes, confirming that the work function of mesoporous TiO_2_ was modified by Ag deposition. We conclude that Ag metal loading can adjust the band bending and Fermi level of an anatase TiO_2_ surface, leading to the improvement in the reactivity on the surface of TiO_2_, thus promoting the accessibility of photoelectrons at the conduction band where H^+^ is reduced to hydrogen gas. On the other hand, Figure 9a,b shows that OH^1–*^ intermediate formation required smaller step change of Δ*G* when Ag nanoparticle was deposited on TiO_2_. This indicates the higher probability of paracetamol oxidation with Ag/TiO_2_.

**Figure 10:**
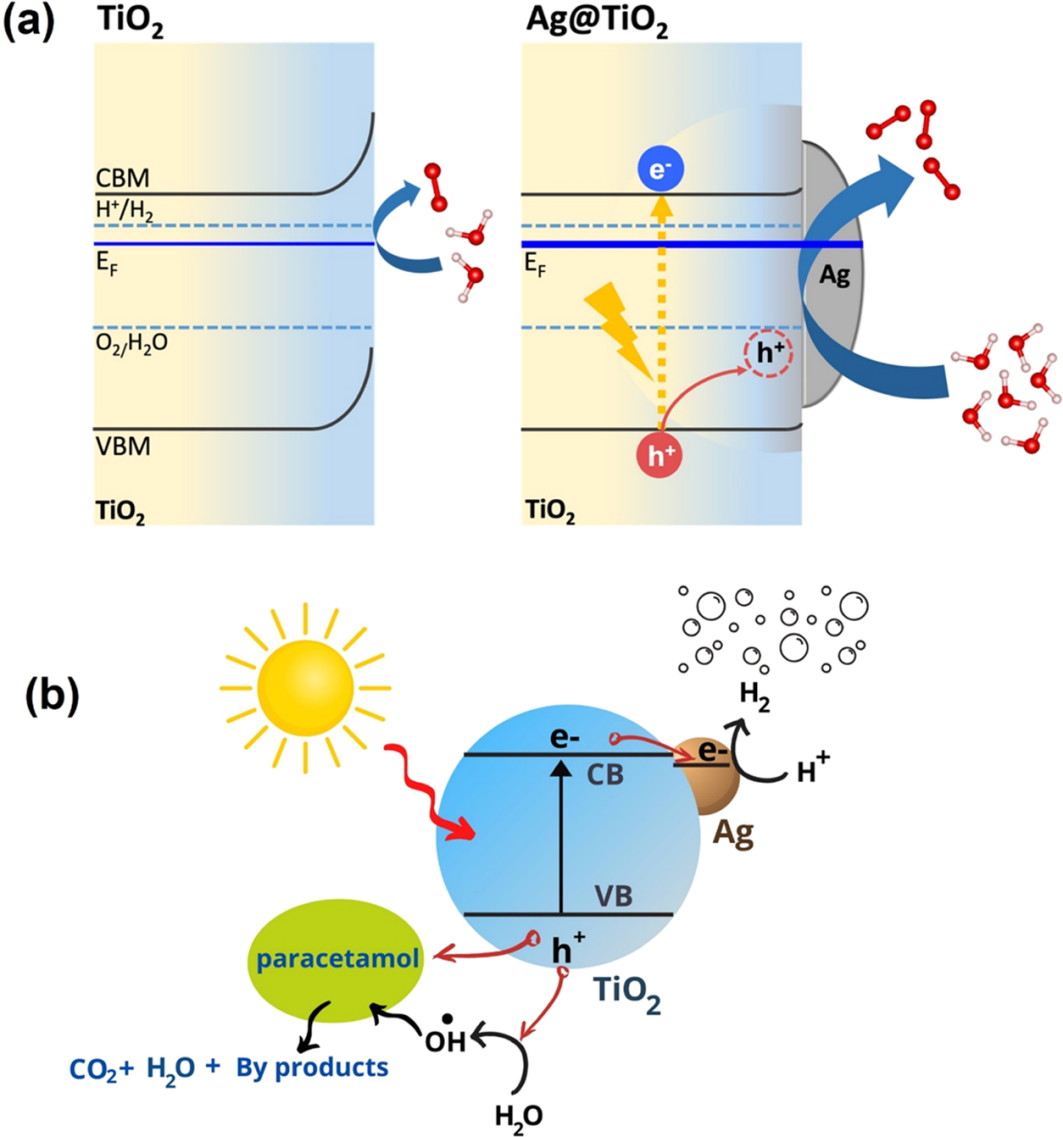
Schematic illustration of (a) energy band diagrams of pristine anatase TiO_2_ and Ag@TiO_2_ and (b) the photocatalytic degradation of paracetamol and hydrogen gas evolution on Ag/TiO_2_ photocatalysts.

Based on the experimental and theoretical results, the process of pharmaceutical water pollutant degradation and hydrogen gas generation at Ag/TiO_2_ photocatalysts is schematically explained in Figure 10. Note that examining paracetamol degradation pathways is not the focus of our work; however, it is worth investigating the byproducts and their potentially toxic nature.^88−90^

## Conclusions

Mesoporous TiO_2_ was synthesized via a gram-scale chemical route using the P123 surfactant as a template. Presynthesized Ag nanoparticles were successfully coated onto TiO_2_, and structural and textural properties of the resultant Ag/TiO_2_ composite were examined. This modified Ag/TiO_2_ showed higher photocatalytic performance for paracetamol degradation and H_2_ production compared with pristine TiO_2_. The high activity of mesoporous Ag/TiO_2_ can be attributed to several factors: (a) strong inhibition of the e^–^–h^+^ recombination due to the Schottky barrier formation at the TiO_2_–Ag interface,^91^ (b) hydroxyl group formation facilitating pollutant adsorption on the TiO_2_ surface, and (c) extended visible-light activity. Over this most active catalyst, 100% degradation of paracetamol was reached after only 90 min with 98% total organic content (TOC) abatement and 1729 μmol H_2_ g^–1^ h^–1^ was achieved for hydrogen generation, which largely exceeds that obtained over pristine mesoporous TiO_2_ (875 μmol H_2_ g^–1^ h^–1^). Moreover, 1 wt % Ag/mesoporous TiO_2_ was stable, and Ag effectively optimized the Fermi level of the TiO_2_ surface for higher reactivity. Thus, Ag/TiO_2_ is an attractive photocatalyst candidate for tandem environmental remediation and hydrogen generation under solar irradiation. Conversely, comparative tests with other dopants such as N- and Ln-doped TiO_2_ showed enhanced performance in either pollutant degradation or hydrogen evolution over pristine TiO_2_, but not for both processes. These results suggest that for further research on analyzing the surface functionality of photocatalysts and determining energy levels with respect to the HER and OER potential, charge transfer resistance at semiconductor catalyst/electrolyte interfaces will help to further optimize doped TiO_2_ for effective simultaneous photocatalytic reactions. The gram-scale-synthesized Ag nanoparticle-doped TiO_2_ photocatalyst powder from this work can be coated on substrates to facilitate photocatalyst recycling for batch reactors.

## Supporting Information Available

Photography of TiO_2_ and Ag/TiO_2_ photocatalysts, TEM images, Raman spectra, optical bandgap results, BET results, and UPS results (PDF)

## Abbreviations Used

BID: barrier-discharge ionization detector
CBM: conduction band maximum
DFT: density functional theory
GC: gas chromatography
GGA: generalized gradient approximation
HRTEM: high-resolution transmission electron microscopy
JCPDS: joint committee on powder diffraction standards
LSPR: localized surface plasmon resonance
OER: oxygen evolution reaction
Ov: oxygen vacancies
PAW: projector-augmented wave method
PBE: Perdew–Burke–Ernzerhof
PPCP: pharmaceutical and personal care products
TEOA: triethanolamine
TOC: total organic carbon
UPS: ultraviolet photoelectron spectroscopy
VASP: Vienna ab initio simulation package
VBM: valence band maximum
XPS: X-ray photoelectron spectroscopy
XRD: X-ray diffraction
