# Supplementary material for: Facile Synthesis of Gram-Scale Mesoporous Ag/TiO2 Photocatalysts for Pharmaceutical Water Pollutant Removal and Green Hydrogen Generation
Source: ACS Omega. 2022 Dec 28;8(1):1249–61. doi: 10.1021/acsomega.2c06657 (PMC9835632; doi:10.1021/acsomega.2c06657)
Supplement: Supplementary file 1 — ao2c06657_si_001.pdf [file ao2c06657_si_001.pdf]

Supporting information

Facile Synthesis of Gram-scale Mesoporous Ag/TiO_2 Photocatalysts for Pharmaceutical Water Pollutant Removal and Green Hydrogen Generation

Yassine Cherif,[1] Hajer Azzi,[1,2]* Kishore Sridharan,[3] Seulgi Ji,[4] Heechae Choi,[4] Michael G. Allan,[5] Sihem Benaissa,[2] Karima Bendahou,[2] Lois Afua Damptey,[6] Camila Silva Ribeiro,[6] Satheesh Krishnamurthy,[6] Sanjay Nagarajan,[7] M. Mercedes Maroto-Valer,[8] Moritz F. Kuehnel,[5,9] Sudhagar Pitchaimuthu[8]*

1. Laboratoire de Catalyse et Synthèse en Chimie Organique BP 119, Université de Tlemcen, Tlemcen 13000, Algeria

2. Université d'Ain Témouchent, Institut des Sciences et de la Technologie, BP 284, 46000 Ain Témouchent, Algeria

3. Department of Nanoscience and Technology, School of Physical Sciences, University of Calicut, P.O. Thenhipalam 673635, India

4. Theoretical Materials & Chemistry Group, Institute of Inorganic Chemistry, University of Cologne, Greinstr. 6, 50939, Cologne, Germany

5. Department of Chemistry, Swansea University, Singleton Park, Swansea, United Kingdom

6. School of Engineering & Innovation, The Open University, Walton Hall, Milton Keynes, MK7 6AA, United Kingdom

7. Department of Chemical Engineering, University of Bath, Bath, United Kingdom

8. Research Centre for Carbon Solutions, Institute of Mechanical and Processing Engineering, School of Engineering & Physical Science, Heriot-Watt University Edinburgh, EH14 4AS, United Kingdom

9. Fraunhofer Institute for Wind Energy Systems IWES, Am Haupttor 4310, 06237 Leuna, Germany

Contents

Figure S1. Photography of gram scale (a) mesoporous TiO_2 powder and (b) 1 wt% Ag nanoparticles coated mesoporous TiO_2 powder.

Figure S2. Photocatalytic degradation tests of paracetamol under natural solar light irradiation using different photocatalysts (TiO_2, Ag/TiO_2, N-TiO_2 and La-TiO_2).

Figure S3. HRTEM images of pristine TiO_2 at different magnification scale (a) 50 nm and (b) 10 nm.

Figure S4. HRTEM images of Ag (1 wt%) decorated TiO_2 nanoparticles at 2 nm scale.

Figure S5. Raman spectra of prisitine and Ag nanoparticles (0.5 and 1 wt%) coated TiO_2. The results were compared with N-TiO_2 and La-TiO_2 powders.

Figure S6. Optical bandgap energy of N and La doped TiO_2.

Figure S7. Valence band and conduction band poisition of pristine and pristine and Ag nanoparticles coated TiO_2 powders.

Table S1. Textural properties and band gap energies of pure and doped mesoporous TiO_2

S1. Synthesised powder:

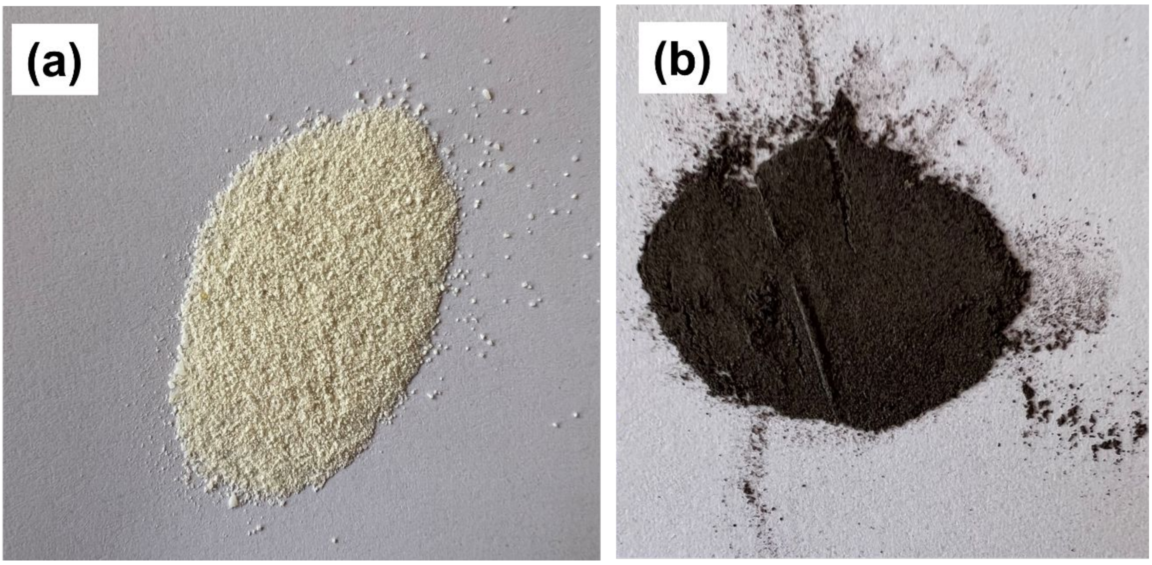

Figure S1. Photography of gram scale (a) mesoporous TiO_2 powder and (b) 1 wt% Ag nanoparticle-coated mesoporous TiO_2 powder.

S2. Experimental set up

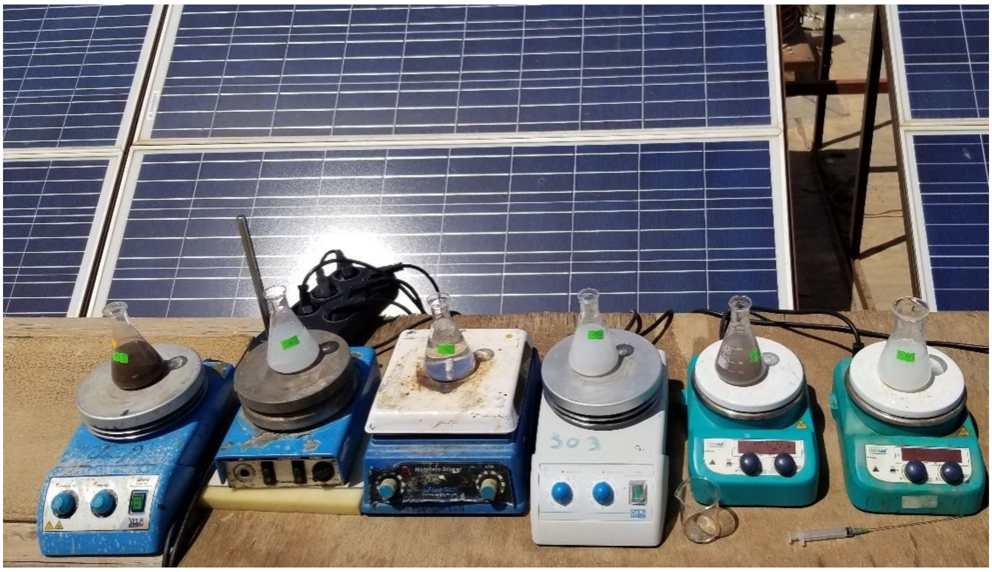

Figure S2. Photocatalytic degradation tests of paracetamol under natural solar light irradiation using different photocatalysts (TiO_2, Ag/TiO_2, $N\text{-}TiO_2$ and $La\text{-}TiO_2$).

S3. Surface morphology analysis

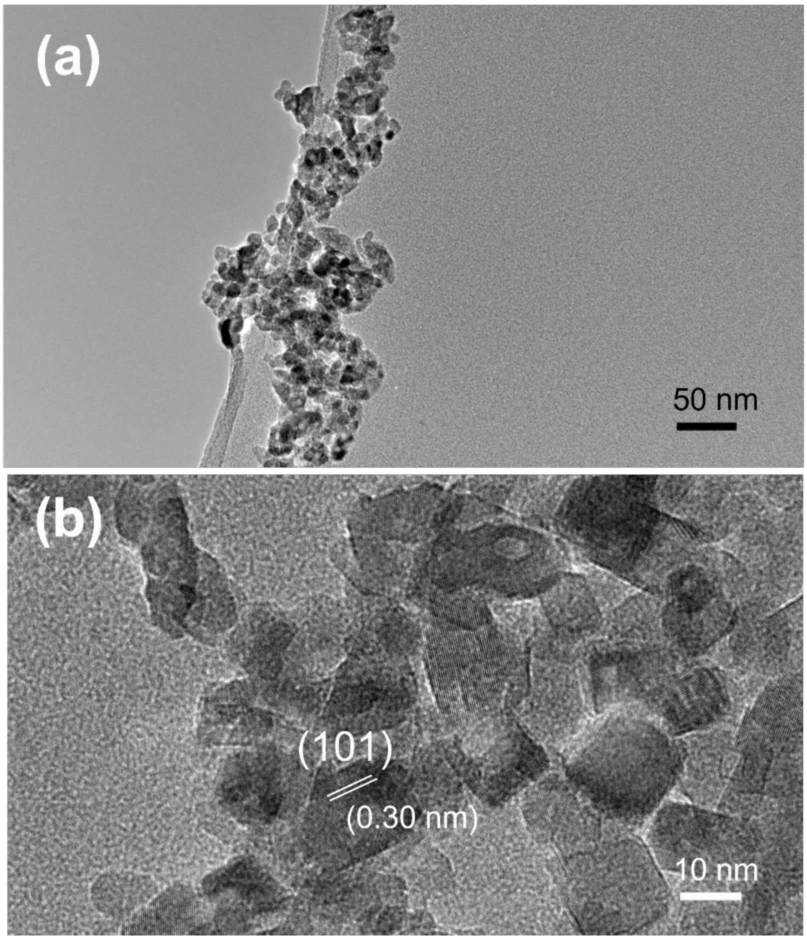

Figure S3. HRTEM images of pristine TiO_2 at different magnification scales (a) 50 nm and (b) 10 nm.

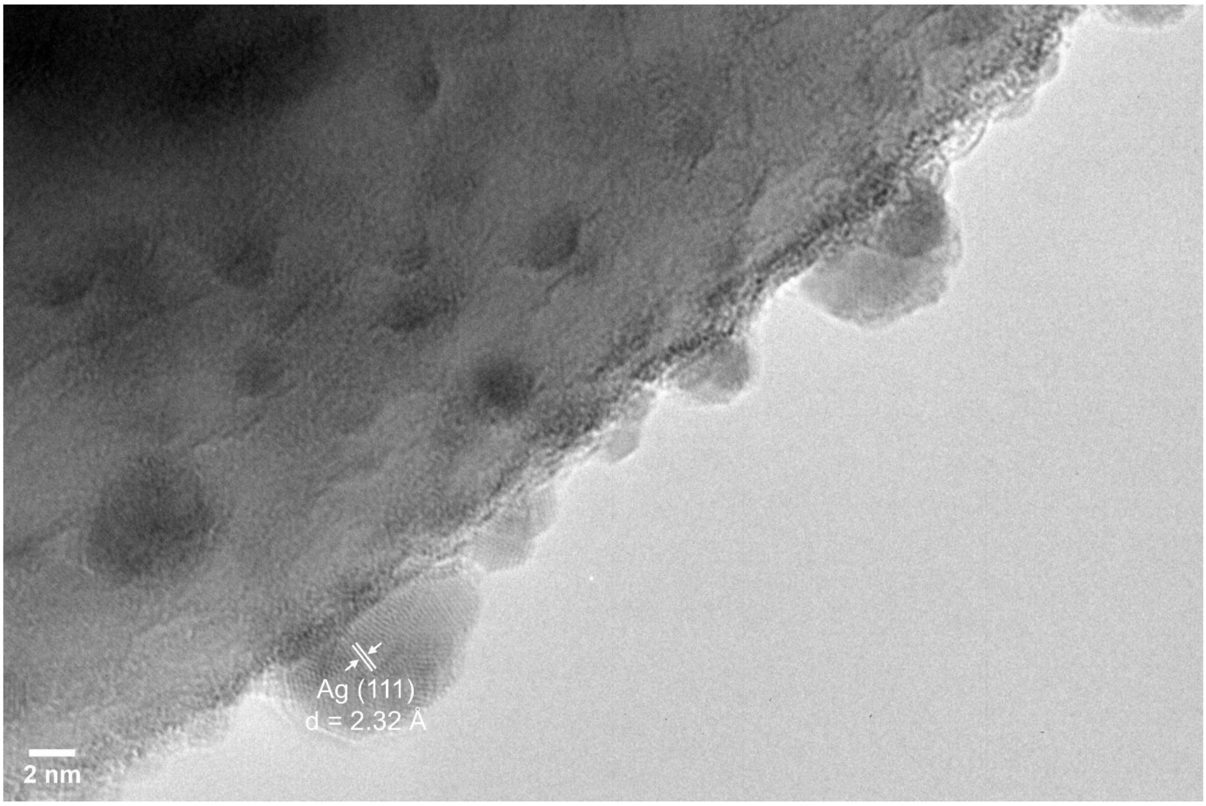

Figure S4. HRTEM images of Ag (1 wt%) decorated TiO_2 nanoparticles at 2 nm scale.

S4. Chemical environment- C1s

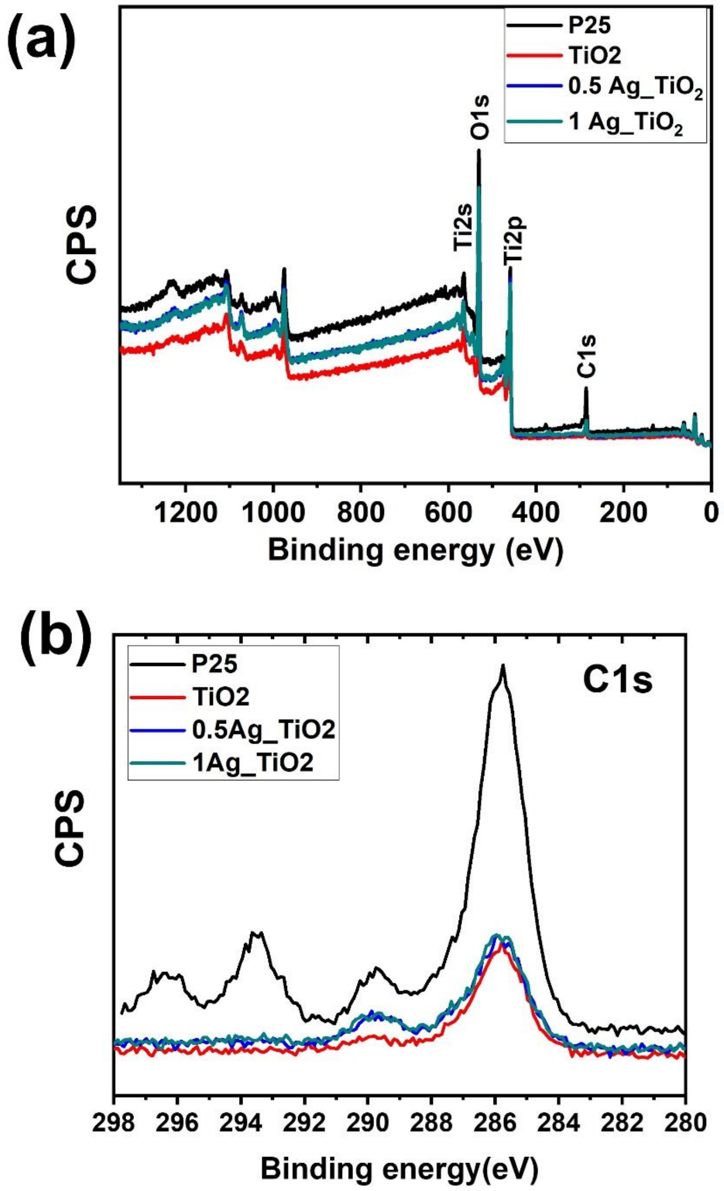

Figure S5. XPS results of pristine and Ag nanoparticles decorated TiO_2. (a) Survey spectra and (b) C1s core spectra of pristine and Ag nanoparticle-coated (0.5 and 1 wt%) TiO_2. The results were compared with commercial P25 TiO_2.

S5. Raman spectra

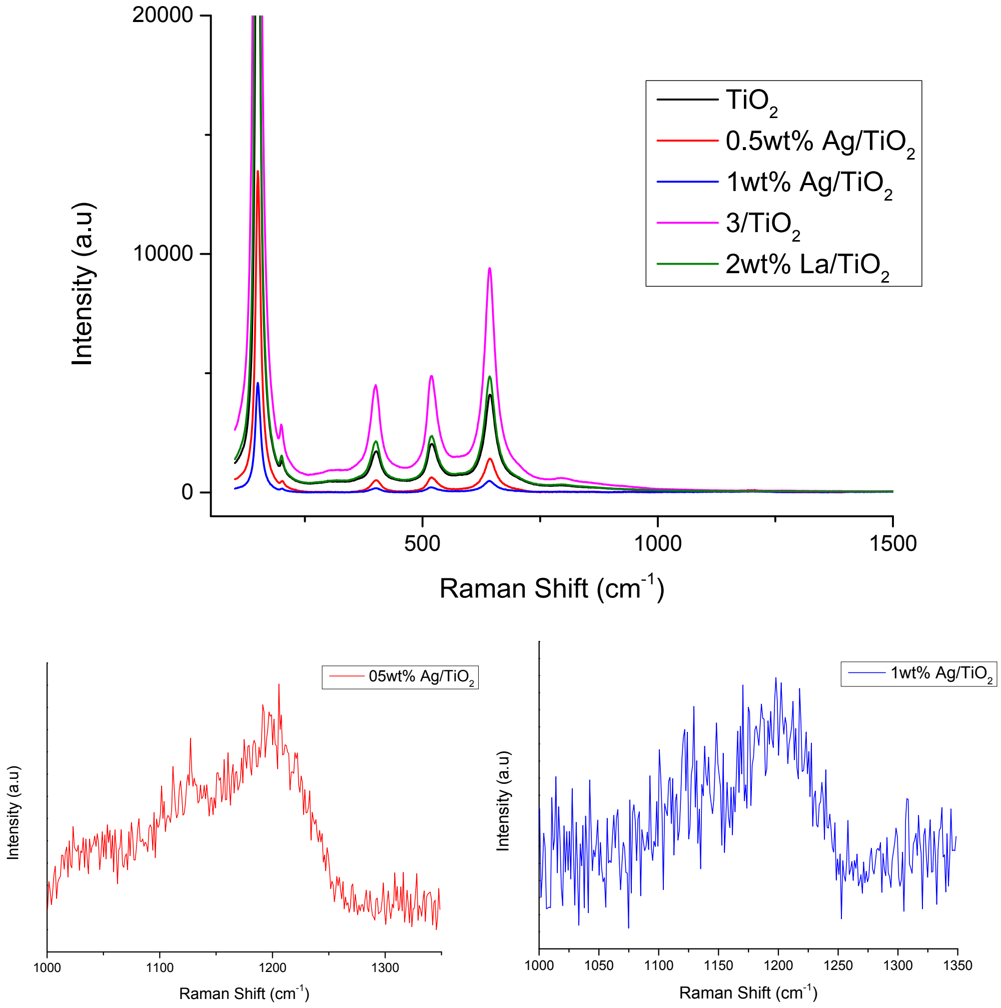

Figure S6. Raman spectra of pristine and Ag nanoparticle-coated (0.5 and 1 wt%) TiO_2. The results were compared with N-TiO_2 and La-TiO_2 powder.

Raman spectra of pristine and Ag nanoparticle-coated mesoporous TiO_2 are shown in Figure S5. Pristine TiO_2 spectrum showed high intensity Raman band at 149 cm^{-1} and four other Raman bands at 201, 401, 519 and 643 cm^{-1}, which could be attributed to Eg , B1g , A1g (B1g), and Eg Raman modes of anatase TiO_2, respectively[1]. This result is in good agreement with the XRD characterization. For silver-doped TiO_2, the Eg mode of TiO_2 was observed to shift from 149 cm^{-1} to 150 cm^{-1}, and from 201 cm^{-1} to 202 cm^{-1}, which could be ascribed to

the metal-support interaction, which is well consistent with XPS characterization. On the other hand, a low-intensity Raman band was observed at 1210 cm^{-1} which could be attributed to the bending vibration of carboxylate group, δ(COO) of the citrate reducing agent probably adsorbed on silver nanoparticles.[2]

S6. Optical analysis

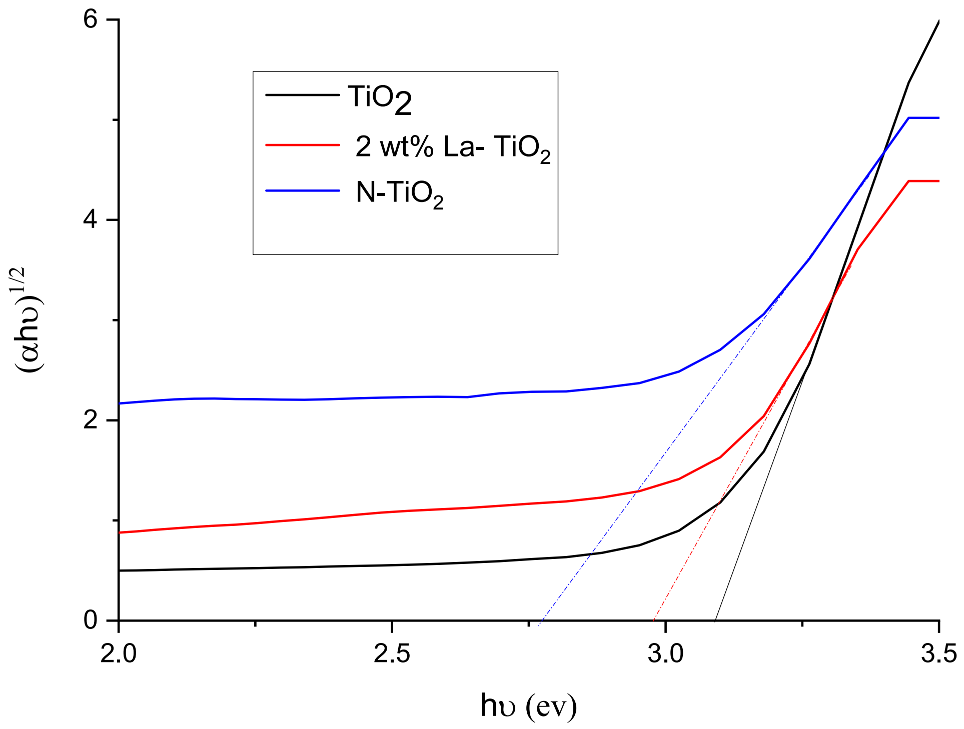

Figure S7. Optical bandgap energy of pristine, N and La-doped TiO_2.

The optical bandgap energy of pristine mesoporous TiO_2, nitrogen-doped TiO_2 and La-doped TiO_2 are estimated from Figure S5 and found to be 3.1 eV, 2.75 eV and 2.97 eV, respectively.

S7. Work function

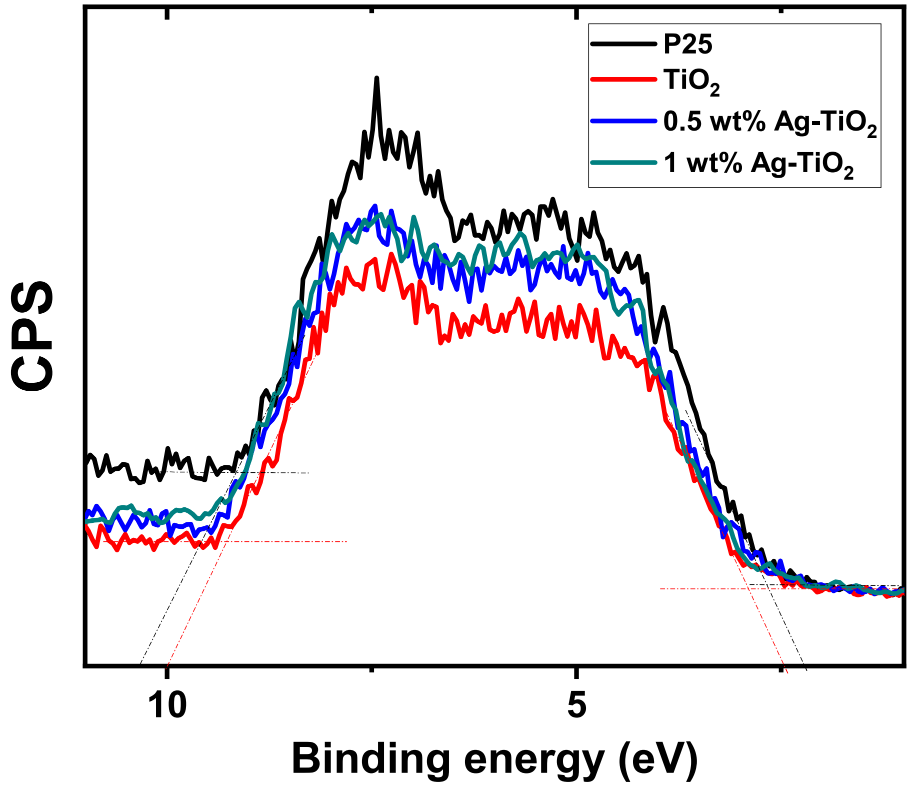

Figure S8. Ultraviolet photoelectron spectroscopy (UPS) of pristine and Ag nanoparticle-coated TiO_2 powder. The results were compared with commercial TiO_2 powder.

The UPS results from Figure S6 ensure that the valence band (VB) position of mesoporous TiO_2 is slightly higher (2.92 eV) than that of commercial P25 TiO_2 (2.62 eV). However, there is no change in VB position after Ag nanoparticle decoration. However, a small shift in onset position at higher binding energy position at Ag-TiO_2 samples indicates the conduction band edge modification by Ag nanoparticles coating on TiO_2 surface.

The work function of the semiconductor can estimated using the following equation, [3]

$$\varphi = hv - (E_{\mathrm{FE}} - E_{\mathrm{onset}}) \quad (1)$$

where, hv = 21.2 eV (He I source), E_{onset} is the onset of the secondary emission, and E_{FE} is the Fermi edge. The onset of the secondary emission (E_{onset}) was determined by extrapolating two solid lines from the background and straight onset. In this equation, the changes in onset position indicates the work function variation. The work function modification at Ag/TiO_2

anticipated to facilitate the photoelectron flow from TiO_2 to surface Ag which accelerate the photocatalytic activity for pollutant degradation.[4]

Table S1. Textural properties and band gap energies of pure and doped mesoporous TiO_2

Catalyst	$S_{BET}(m^2/g)$	Pore Size (nm)	Pore volume (cm^3/g)
TiO_2	102	11.01	0.325
0.5wt%Ag/TiO_2	85	11.65	0.321
1wt%Ag/TiO_2	90	11.64	0.335

References:

1. Zhou, W.; Sun, F.; Pan, K.; Tian, G.; Jiang, B.; Ren, Z.; Tian, C.; Fu, H., Well-ordered large-pore mesoporous anatase TiO2 with remarkably high thermal stability and improved crystallinity: preparation, characterization, and photocatalytic performance. *Advanced Functional Materials* **2011,** *21* (10), 1922-1930.
2. Pandoli, O.; Martins, R.; Romani, E.; Paciornik, S.; Maurício, M.; Alves, H.; Pereira-Meirelles, F.; Luz, E.; Koller, S.; Valiente, H., Colloidal silver nanoparticles: an effective nano-filler material to prevent fungal proliferation in bamboo. *RSC advances* **2016,** *6* (100), 98325-98336.
3. Ishii, H.; Sugiyama, K.; Ito, E.; Seki, K., Energy Level Alignment and Interfacial Electronic Structures at Organic/Metal and Organic/Organic Interfaces. *Advanced Materials* **1999,** *11* (8), 605-625.
4. Choi, J.; Sudhagar, P.; Lakshmipathiraj, P.; Lee, J. W.; Devadoss, A.; Lee, S.; Song, T.; Hong, S.; Eito, S.; Terashima, C.; Han, T. H.; Kang, J. K.; Fujishima, A.; Kang, Y. S.; Paik, U., Three-dimensional Gd-doped TiO2 fibrous photoelectrodes for efficient visible light-driven photocatalytic performance. *RSC Advances* **2014,** *4* (23), 11750-11757.
